# Using bioinformatics and systems biology methods to identify the mechanism of interaction between COVID-19 and nonalcoholic fatty liver disease

**DOI:** 10.1097/MD.0000000000033912

**Published:** 2023-06-09

**Authors:** Wenbo Dong, Yan Jin, Hongshuo Shi, Xuecheng Zhang, Jinshu Chen, Hongling Jia, Yongchen Zhang

**Affiliations:** a Shandong Traditional Chinese Medicine University, Jinan, China; b Beijing University of Chinese Medicine, Beijing, China; c The Second Affiliated Hospital of Shandong University of Chinese Medicine, Jinan, China; d Affiliated Hospital of Shandong University of Traditional Chinese Medicine, Jinan, China.

**Keywords:** bioinformatics, COVID-19, drug repositioning, Ferroptosis, NAFLD

## Abstract

Nonalcoholic fatty liver disease (NAFLD) is considered a risk factor for severe COVID-19, but the mechanism remains unknown. This study used bioinformatics to help define the relationship between these diseases. The GSE147507 (COVID-19), GSE126848 (NAFLD), and GSE63067 (NAFLD-2) datasets were screened using the Gene Expression Omnibus. Common differentially expressed genes were then identified using a Venn diagram. Gene ontology analysis and KEGG pathway enrichment were performed on the differentially expressed genes. A protein–protein interaction network was also constructed using the STRING platform, and key genes were identified using the Cytoscape plugin. GES63067 was selected for validation of the results. Analysis of ferroptosis gene expression during the development of the 2 diseases and prediction of their upstream miRNAs and lncRNAs. In addition, transcription factors (TFs) and miRNAs related to key genes were identified. Effective drugs that act on target genes were found in the DSigDB. The GSE147507 and GSE126848 datasets were crossed to obtain 28 co-regulated genes, 22 gene ontology terms, 3 KEGG pathways, and 10 key genes. NAFLD may affect COVID-19 progression through immune function and inflammatory signaling pathways. CYBB was predicted to be a differential ferroptosis gene associated with 2 diseases, and the CYBB-hsa-miR-196a/b-5p-TUG1 regulatory axis was identified. TF-gene interactions and TF-miRNA coregulatory network were constructed successfully. A total of 10 drugs, (such as Eckol, sulfinpyrazone, and phenylbutazone) were considered as target drugs for Patients with COVID-19 and NAFLD. This study identified key gene and defined molecular mechanisms associated with the progression of COVID-19 and NAFLD. COVID-19 and NAFLD progression may regulate ferroptosis through the CYBB-hsa-miR-196a/b-5p-TUG1 axis. This study provides additional drug options for the treatment of COVID-19 combined with NAFLD disease.

## 1. Introduction

Novel coronavirus disease 2019 (COVID-19) pneumonia is a highly pathogenic viral infection caused by severe acute respiratory syndrome coronavirus 2 (SARS-CoV-2). Typical clinical symptoms include moderate fever (37.3–38.0°C), cough, fatigue, diarrhea and myalgia. Additional symptoms can include shortness of breath, altered consciousness, blurry vision, headache, sore throat, runny nose, chest pain, nausea, and vomiting.^[1,2]^ COVID-19 rapidly spread around the world and on March 11, 2020, the World Health Organization declared the disease a pandemic that was a serious danger to human health and a heavy burden on society.^[3]^ As of September 2022, the COVID-19 pandemic had infected more than 600 million people worldwide and killed more than 6.5 million people (https://coronavirus.1point3acres.com/). Studies have found that cytokine storm plays a key role in the progression of COVID-19,^[4]^ and the SARS-CoV-2 virus can transcribe 9 subgenomic RNAs that encode structural proteins able to interfere with the host’s innate immune response function.^[5]^

Nonalcoholic fatty liver disease (NAFLD) is one of the most common causes of chronic liver disease and is primarily characterized by the accumulation of excess lipids in the liver.^[6,7]^ Obesity is a key factor in the development of NAFLD.^[8]^ Some studies have also found that particular tumor necrosis factors are also closely related to the progression of NAFLD.^[9,10]^ At present, effective NAFLD treatments and interventions have not yet been developed.^[11]^

Recent studies have identified a potential association between NAFLD and both the probability of testing positive for SARS-CoV-2 and the severity and mortality of COVID-19.^[12]^ NAFLD patients are in a state of chronic inflammation, which can increase the risk of SARS-CoV-2 infection as a result of impaired immune function.^[13]^ A recent retrospective study assessing NALFD status in 202 COVID-19 patients showed that liver injury was present in 50% and 75.2% of patients during admission and hospitalization, respectively.^[14]^ SARS-CoV-2 in gut lumen could translocate to the liver via portal flow and induce a direct damage due to active viral replication in hepatic cells. In terms of gene expression, Hepatocellular hypoxia in chronic liver diseases in COVID-19-infected patients might lead to increased expression of ACE2 receptors, and hypoxia-inducible factors (HIFs).^[13]^ While the correlation between COVID-19 and NAFLD has become more evident, the biological mechanism for this relationship requires further exploration.

Ferroptosis is a novel form of programmed cell death with a complex cell signaling mechanism characterized by iron-dependent lipid peroxide accumulation and loss of activity of the lipid repair enzyme glutathione peroxidase 4 (GPX4).^[15,16]^ Ferroptosis is mostly associated with neoplastic disease.^[17]^ However, iron filariasis has now been shown to have an important pathogenic role in a variety of systemic diseases, such as chronic liver disease and COVID-19.^[18–20]^

**Figure 1.**
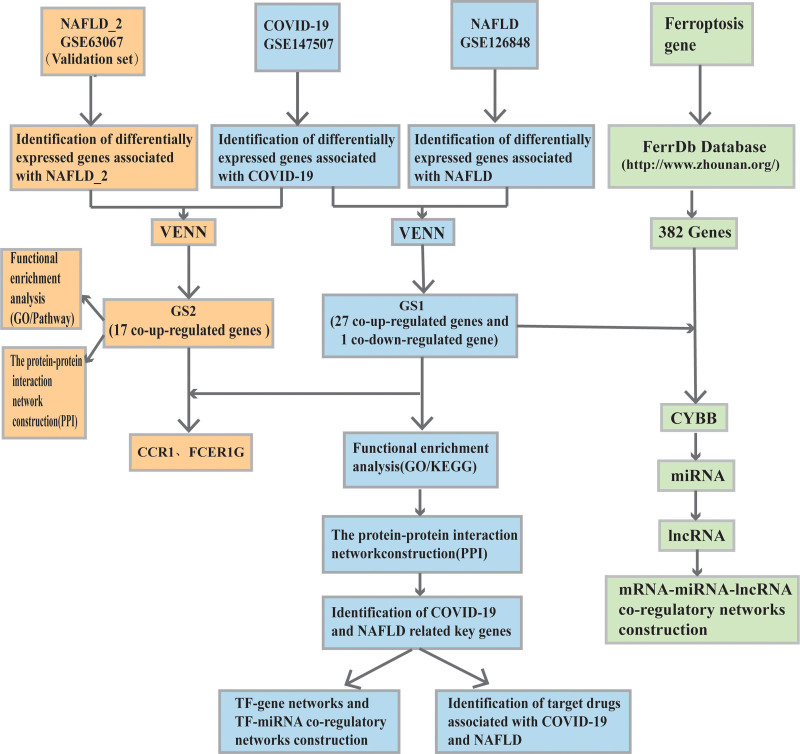
Workflow chart of this study. COVID-19 = coronavirus disease, GO = gene ontology, KEGG = Kyoto Encyclopedia of Genes and Genomes, NAFLD = nonalcoholic fatty liver disease, PPI = protein-protein interaction.

Bioinformatics is a comprehensive application of information science, statistics, computer science, and other technologies to process and analyze large amounts of data and study biological problems. In recent decades, bioinformatics has become a new method of analysis for the field of life sciences and provided some important research results.^[21]^ Keying et al^[22]^used this approach to analyze genetic samples, explored relevant mechanisms after human bronchial tube infection with the new coronavirus, and found that CXCL10 expression correlated with SARS-CoV-2 virus infection. Mamber et al and Hue et al^[23,24]^ confirmed that CXCL10, an interferon protein, increases CXCL10 and CXCL10/IP-10 expression in response to SARS-CoV-2 infection. CK2 kinase activity is also elevated in cells infected with SARS-CoV-2 and Miranda et al^[25]^ validated this by applying CIGB-300, an antagonist of CK2 kinase activity, and analyzing the results using bioinformatics. CIGB-300 treatment resulted in the emergence of a highly specific immune response to SARS-CoV-2 infection. Zhang et al^[26]^ analyzed microRNA and mRNA expression profiles and found that miR-128 and miR-130b could regulate the MAPK pathway in NAFLD mice. Thus, wider use of bioinformatics approaches, combined with other strategies and tools, may provide a deeper understanding of the underlying association between NAFLD and COVID-19. The flow chart for this study is presented in Figure 1.

## 2. Materials and methods

### 2.1. Tools

The GSE147507 (COVID-19), GSE126848 (NAFLD), and GSE63067 (NAFLD-2) datasets were selected from the Gene Expression Omnibus database (https://www.ncbi.nlm.nih.gov). Valid differentially expressed genes (DEGs) were screened using the NetworkAnalyst (https://www.networkanalyst.ca) database and EXCEL. The Omicshare cloud platform (https://www.omicshare.com) was used to make volcano and cluster analysis maps. Jvenn (http://jvenn.toulouse.inra.fr/) was used to select crossover genes. Gene ontology (GO) and Kyoto Encyclopedia of Genes and Genomes (KEGG) pathway enrichment were performed using the STRING platform (https://cn.string-db.org), Metascape (http://metascape.org/), and DAVID website (https://david.ncifcrf.gov), respectively. Protein–protein interaction (PPI) network visualization was conducted using the STRING platform. Bioinformatics online platform for ROC curve analysis (https://www.bioinformatics.com.cn). Cytoscape was used to screen key genes and visualize the network. Ferroptosis-related genes from the FerrDb database (http://www.zhounan.org/). Predicting miRNAs and lncRNAs using the Starbase database (http://starbase.sysu.edu.cn). The NetworkAnalyst database (https://www.networkanalyst.ca/) was used to identify transcription factors (TFs) and TF-miRNA action relationships associated with key genes. The DSigDB gene sets (https://maayanlab.cloud/Enrichr/) was used to identify potential drugs.

### 2.2. Data collection

The GSE147507, GSE126848, and GSE63067 datasets were obtained from the Gene Expression Omnibus database. GSE147507 included 2 COVID-19 lung biopsy samples and 2 healthy human samples. GSE126848 included 31 NAFLD patients with different degrees of fatty liver and 14 healthy controls. GSE63067 included 9 NAFLD patients and 7 healthy controls.

### 2.3. Identification of DEGs in NAFLD and COVID-19 samples

Gene screening was carried out on the GSE126848 and GSE147507 datasets according to adj.*P* ≤ .05 and ∣ LogFC∣≥1. Using the screening conditions, 2 groups of effective DEGs were identified, and the online Omicshare cloud platform was used for data visualization and the construction of volcano and cluster analysis plots. Valid DEGs from the 2 datasets were stored in the online platform, jvenn, and the intersection of the genes was used to obtain common DEGs, defined as GS1.

### 2.4. Gene ontology and pathway enrichment analysis of GS1

GO can provide annotations of genes, gene products, and sequences, involving biological processes, molecular functions, and cellular components, and conceptually integrate data for widespread use.^[27]^ The DAVID bioinformatics resource is used to provide corresponding functional interpretations of large amounts of genetic data.^[28]^ GO and KEGG pathway enrichment analysis was performed on DEGs using the STRING website and DAVID platform, respectively. The critical value of significant gene analysis was set as *P* ≤ .05.

### 2.5. Construction of PPI networks and identification of key genes

Twenty-eight DEGs were imported into the STRING platform for PPI network construction. The Cytoscape plug-in cytoHubba, one of the most successful network biology analysis and visualization tools,^[29]^ was used to screen key genes and the MCC algorithm was used to obtain the top 10 key genes.

### 2.6. GSE63067 validates GS1 analysis results

To validate the results, we screened the cross gene of GSE147507 and GSE63067 and defined it as GS2. The function enrichment GO was analyzed, and the network diagram was constructed. In addition, we used ROC curve analysis to determine the diagnostic value of the predicted outcomes.

### 2.7. Analysis of the correlation between 2 diseases and ferroptosis

Ferroptosis-related genes were obtained from the FerrDb database (http://www.zhounan.org/). GS1 was used to screen for differentially expressed genes associated with ferroptosis. We use the Starbase database to predict the upstream regulatory genes miRNA and long-stranded non-coding RNAs (lncRNAs) associated with them and construct a network of relationships.

### 2.8. Identification of TF genes associated with DEGs

TFs can transcribe genetic information and regulate the expression of related genes. Abnormal TF function is associated with many diseases and TFs are often used as therapeutic targets.^[30]^ The TF-Gene interactions function of the NetworkAnalyst database can be used to search for TFs that are related to key genes. Key genes are imported into the input box of the NetworkAnalyst database, the TFs-Gene interaction function is selected, data is gathered from the ENCODE database, and the BETA Minus algorithm is used to obtain the results of interactions between key genes and TFs. A visual network analysis diagram can be used to reflect the expression regulation level of TFs on DEGs.

### 2.9. TF-miRNA synergy analysis

Using the NetworkAnalyst database platform, interaction information from the RegNetwork database was collected, and the network associations between TF-miRNAs and key synergistic genes were visualized to predict gene transcriptional regulation.^[31]^

### 2.10. Prediction of drug candidates

DSigDB can link gene expression with drugs/compounds for drug repurposing and translational research. DSigDB currently holds 22,527 gene sets, consists of 17,389 unique compounds covering 19,531 genes.^[32]^ We accessed the DSigDB database through the online website (https://maayanlab.cloud/Enrichr/). Target gene were imported into the network platform, and candidate drugs for the treatment of the NAFLD and COVID-19 were identified using a systematic algorithm.

## 3. Results

### 3.1. DEG expression in NAFLD and COVID-19

**Figure 2.**
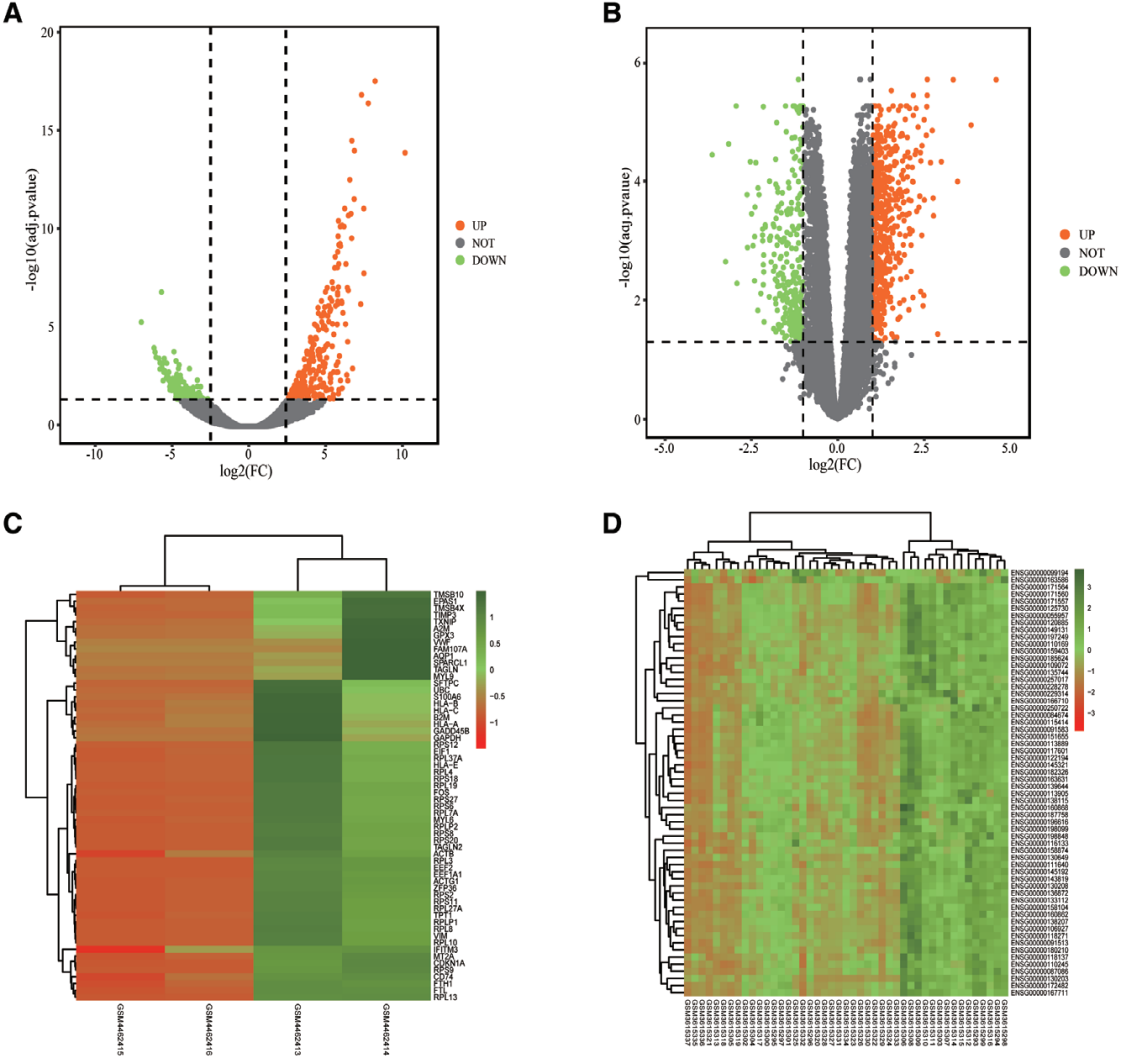
Differentially expressed genes. (A) Differentially expressed genes (DEGs) were identified in GSE147507. (B) Differentially expressed genes (DEGs) were identified in GSE126848. Gray dots represent genes that did not change expression, orange dots represent up-regulated differential genes, and green dots represent down-regulated genes. (C) GSE14750 DEGs heatmap analysis. (D) GSE126848 DEGs heatmap analysis. The top 60 DEGs with the most obvious differences were screened by Excel. Red represents up-regulated gene expression and green represents down-regulated gene expression. DEGs = differentially expressed genes.

The GSE147507 dataset contained 2 COVID-19 lung biopsy samples and 2 healthy human samples with a total of 14,524 genes. Of these, 495 valid differences were screened with a critical value of adj.*P* ≤ .05 and ∣ LogFC∣≥1 DEGs, including 267 up-regulated genes and 228 down-regulated genes. The GSE126848 dataset included 31 NAFLD patients with different degrees of fatty liver and 14 healthy controls. A total of 12,507 genes were found. With a critical value of adj.P ≤ .05 and ∣ LogFC∣≥1, 886 effective DEGs were screened, of which 576 were up-regulated and 310 were down-regulated. The GSE63067 dataset contained 9 samples of NAFLD patients and 7 samples of healthy individuals, and valid genes were obtained for validation according to P ≤ .05 and ∣ LogFC∣≥1 screening criteria (Table 1). Volcano plots obtained from effective gene expression screening of NAFLD and COVID-19 are shown in Figure 2A and B. Cluster heatmaps of NAFLD and COVID-19 gene expression profiles are shown in Figure 2C and D.

**Table 1:** Overview of the COVID-19, NAFLD, and NAFLD_2 datasets obtained from the GEO database.

Disease	GSE number	Platform	Sample	Screened genes (N)	Up regulated DEGs (N)	Down regulated DEGs (N)
COVID-19	GSE147507	GPL18573	2 case groups and 2 controls	495	267	228
NAFLD	GSE126848	GPL18573	31 case groups and 14 controls	886	576	310
NAFLD_2	GSE63067	GPL570	9 case groups and 7 controls	139	112	27

COVID-19 = coronavirus disease, DEGs = differentially expressed genes, NAFLD = nonalcoholic fatty liver disease.

### 3.2. Identification of co-differential genes

**Figure 3.**
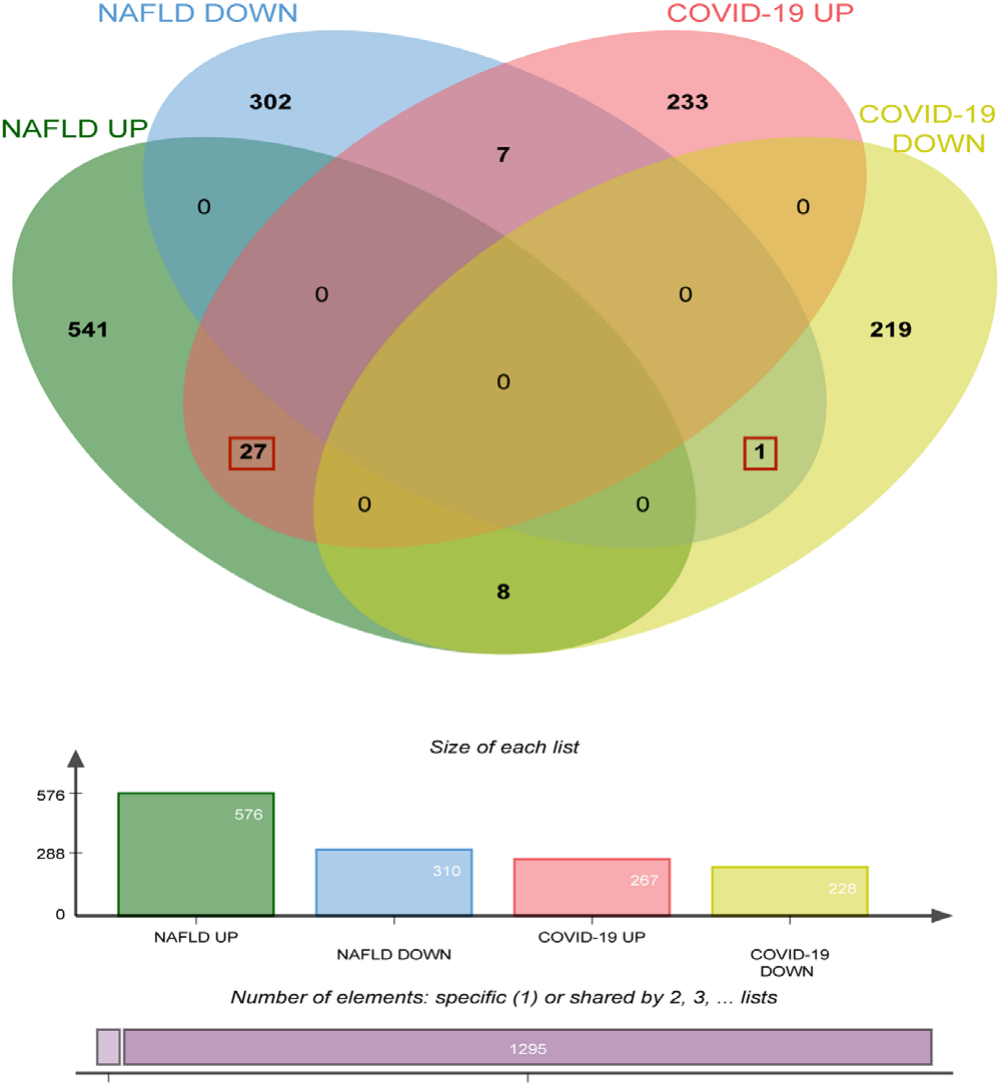
Venn diagram of common crossover DEGs in the GSE147507 and GSE126848. Green represents up-regulated NAFLD genes, blue represents down-regulated NAFLD genes, red represents up-regulated COVID-19 genes, and yellow represents down-regulated COVID-19 genes. COVID-19 = coronavirus disease, DEGs = differentially expressed genes, NAFLD = nonalcoholic fatty liver disease.

Effective crossover genes in the GSE147507 (COVID-19) and GSE126848 (NAFLD) datasets were determined using a Venn diagram. A total of 27 co-up-regulated genes and one co-down-regulated gene were obtained (Fig. 3).

### 3.3. Gene ontology (GO) and pathway enrichment analysis (KEGG) of the DEGs

GO function enrichment analysis was performed on 28 DEGs using STRING online software to describe the biological mechanism. The critical value was set to *P* ≤ .05. The results indicated that DEGs were involved in biological processes including bone marrow leukocyte activation, immune response, neutrophil degranulation, exocytosis. The DEGs mediated immune receptor activity and complement receptor activity. The DEGs were primarily enriched in the secretory granule, ficin-1-rich granule, cytoplasmic vesicle, tertiary granule, and cytoplasmic membranes (Table 2).

**Table 2:** Ontological analysis of common COVID-19 and NAFLD DEGs.

Category	Term ID	Term description	*P* value	Proteins
GO biological process	GO:0002274	Myeloid leukocyte activation	7.54E-09	LCP2, SPI1, FCER1G, C3AR1, MMP25, DOK3, SIGLEC14, CR1, CYBB, SIRPB1, LILRB2, MGAM, FPR1
	GO:0006955	Immune response	2.44E-08	LCP2, FCER1G, CCR1, C3AR1, MMP25, DOK3, SIGLEC14, CR1, AIM2, C1QC, HLA-DRB5, CYBB, SIRPB1, LILRB2, LILRA6, MGAM, FPR1
	GO:0002376	Immune system process	.000000123	LCP2, SPI1, FCER1G, CCR1, C3AR1, MMP25, DOK3, SIGLEC14, CR1, AIM2, C1QC, HLA-DRB5, CYBB, SIRPB1, HCAR2, LILRB2, LILRA6, MGAM, FPR1
	GO:0002443	Leukocyte mediated immunity	.000000139	FCER1G, C3AR1, MMP25, DOK3, SIGLEC14, CR1, C1QC, CYBB, SIRPB1, LILRB2, MGAM, FPR1
	GO:0043312	Neutrophil degranulation	.000000139	FCER1G, C3AR1, MMP25, DOK3, SIGLEC14, CR1, CYBB, SIRPB1, LILRB2, MGAM, FPR1
	GO:0032940	Secretion by cell	.000000306	LCP2, FCER1G, CCR1, C3AR1, MMP25, DOK3, SIGLEC14, CR1, CYBB, SIRPB1, LILRB2, MGAM, FPR1
	GO:0006887	Exocytosis	.00000039	FCER1G, CCR1, C3AR1, MMP25, DOK3, SIGLEC14, CR1, CYBB, SIRPB1, LILRB2, MGAM, FPR1
	GO:0002684	Positive regulation of immune system process	.00000211	LCP2, FCER1G, CCR1, C3AR1, CR1, AIM2, C1QC, HLA-DRB5, SIRPB1, HCAR2, LILRB2, FPR1
	GO:0002682	Regulation of immune system process	.00000299	LCP2, CLC, SPI1, FCER1G, CCR1, C3AR1, CR1, AIM2, C1QC, HLA-DRB5, SIRPB1, HCAR2, LILRB2, FPR1
	GO:0006952	Defense response	.00000507	FCER1G, CCR1, C3AR1, MMP25, SIGLEC14, CR1, AIM2, C1QC, HLA-DRB5, CYBB, SIRPB1, LILRB2, FPR1
GO molecular function	GO:0140375	Immune receptor activity	.00000424	FCER1G, CCR1, C3AR1, CR1, LILRB2, CSF2RB, FPR1
	GO:0004875	Complement receptor activity	.0025	C3AR1, CR1, FPR1
GO cellular component	GO:0030667	Secretory granule membrane	5.6E-10	FCER1G, C3AR1, MMP25, DOK3, SIGLEC14, CR1, CYBB, SIRPB1, LILRB2, MGAM, FPR1
	GO:0101003	ficolin-1-rich granule membrane	6.06E-09	FCER1G, DOK3, SIGLEC14, CR1, LILRB2, MGAM, FPR1
	GO:0070820	Tertiary granule	6.56E-08	FCER1G, DOK3, SIGLEC14, CR1, CYBB, LILRB2, MGAM, FPR1
	GO:0030659	Cytoplasmic vesicle membrane	.000000156	FCER1G, C3AR1, MMP25, DOK3, SIGLEC14, CR1, HLA-DRB5, CYBB, SIRPB1, LILRB2, MGAM, FPR1
	GO:0070821	Tertiary granule membrane	.0000247	FCER1G, SIGLEC14, CYBB, LILRB2, MGAM
	GO:0098805	Whole membrane	.0000539	LCP2, FCER1G, C3AR1, MMP25, DOK3, SIGLEC14, CR1, HLA-DRB5, CYBB, SIRPB1, LILRB2, MGAM, FPR1
	GO:0098588	Bounding membrane of organelle	.00051	HS3ST3A1, FCER1G, C3AR1, MMP25, DOK3, SIGLEC14, CR1, HLA-DRB5, CYBB, SIRPB1, LILRB2, MGAM, FPR1
	GO:0005886	Plasma membrane	.0019	LCP2, KCNJ2, FCER1G, CCR1, C3AR1, BASP1, KCNH7, MMP25, DOK3, SIGLEC14, CR1, HLA-DRB5, CYBB, SIRPB1, HCAR2, LILRB2, CSF2RB, MGAM, FPR1
	GO:0016021	Integral component of membrane	.0058	KCNJ2, HS3ST3A1, FCER1G, CCR1, C3AR1, KCNH7, MMP25, SIGLEC14, CR1, HLA-DRB5, CYBB, SIRPB1, HCAR2, LILRB2, LILRA6, CSF2RB, MGAM, FPR1
	GO:0005887	Integral component of plasma membrane	.0085	KCNJ2, FCER1G, CCR1, C3AR1, KCNH7, CR1, CYBB, SIRPB1, LILRB2, CSF2RB

COVID-19 = coronavirus disease, DEGs = differentially expressed genes, NAFLD = nonalcoholic fatty liver disease.

**Figure 4.**
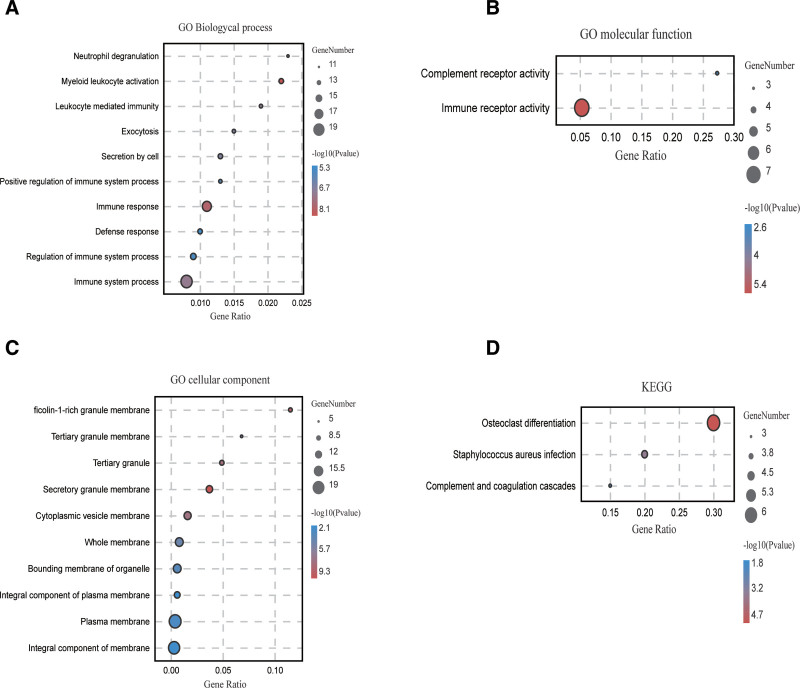
GO and KEGG analysis of COVID-19 and NAFLD related to the common genes. (A) GO enrichment analysis of DEGs based on their biological processes. (B) GO enrichment analysis of DEGs based on their molecular functions. (C) GO enrichment analysis of DEGs based on their cell composition. (D) KEGG pathway enrichment analysis of DEGs. The horizontal axis represents pathway names and the vertical axis represents gene proportions; bubble size represents the number of genes; color gradient from red to blue indicates enrichment significance from high to low. COVID-19 = coronavirus disease, DEGs = differentially expressed genes, GO = gene ontology, KEGG = Kyoto Encyclopedia of Genes and Genomes, NAFLD = nonalcoholic fatty liver disease.

KEGG pathway enrichment analysis was performed on 28 DEGs using the DAVID online network and the threshold was set to *P* ≤ .05. The results indicated that there are 3 KEGG pathways involving osteoclast differentiation, Staphylococcus aureus infection, and complement and coagulation cascade signaling (Table 3). The enriched bubble maps of GO and KEGG are shown in Figure 4.

**Table 3:** pathway enrichment analysis of common COVID-19 and NAFLD DEGs.

Category	Term	Count	%	*P* value	Genes
Pathway	Osteoclast differentiation	6	21.42857143	2.18E-05	LILRA6, SPI1, CYBB, LCP2, LILRB2, SIRPB1
Pathway	Staphylococcus aureus infection	4	14.28571429	4.05E-04	HLA-DRB5, FPR1, C3AR1, C1QC
Pathway	Complement and coagulation cascades	3	10.71428571	.015189147	CR1, C3AR1, C1QC

COVID-19 = coronavirus disease, DEGs = differentially expressed genes, NAFLD = nonalcoholic fatty liver disease.

### 3.4. PPI network construction and screening of key genes

**Figure 5.**
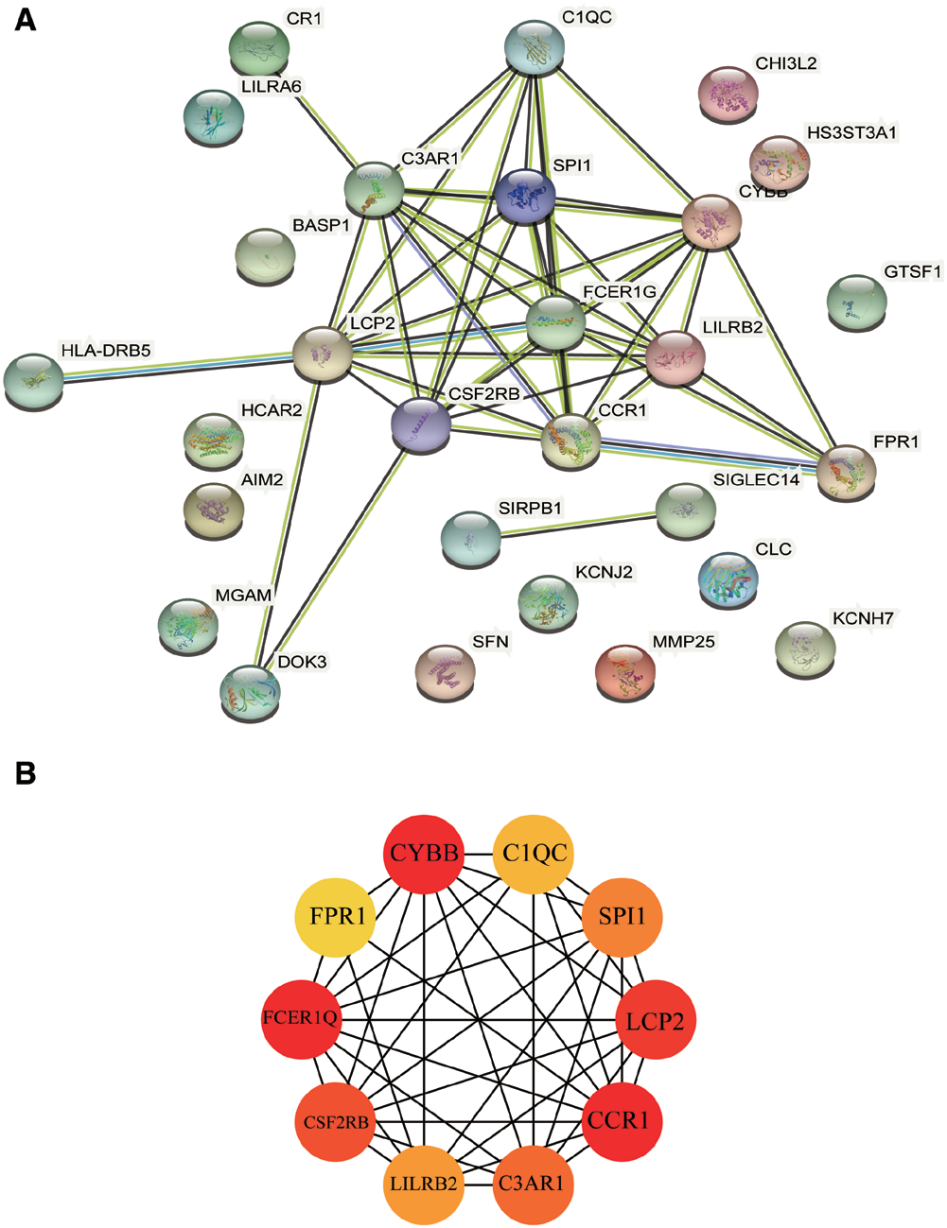
PPI network construction and the top 10 Hub genes (MCC) screening. (A) PPI network of common COVID-19 and NAFLD DEGs. The network has 28 nodes and 44 edges. (B) PPI analysis of the top 10 Hub genes (MCC). The CytoHubba plug-in was used to analyze Hub genes with maximum correlation criterion (MCC), and the top 10 genes were selected. As the color changes from yellow to red, the MCC score increases. COVID-19 = coronavirus disease, DEGs = differentially expressed genes, NAFLD = nonalcoholic fatty liver disease, PPI = protein-protein interaction.

Visual analysis was performed using the STRING platform, and 28 DEGs were imported into various gene list input boxes to construct a PPI network consisting of 28 nodes and 44 edges (Fig. 5A). The Cytoscape cytoHubba plug-in was used to screen key genes (hubs), and the MCC algorithm was used to select the top 10 key gene targets with the highest scores: CYBB, FCER1G, CCR1, LCP2, CSF2RB, C3AR1, SPI1, LILRB2, C1QC, and FPR1 (Fig. 5B).

### 3.5. Validated Cohort: The Differential Genes Analysis in COVID-19 and NAFLD

**Figure 6.**
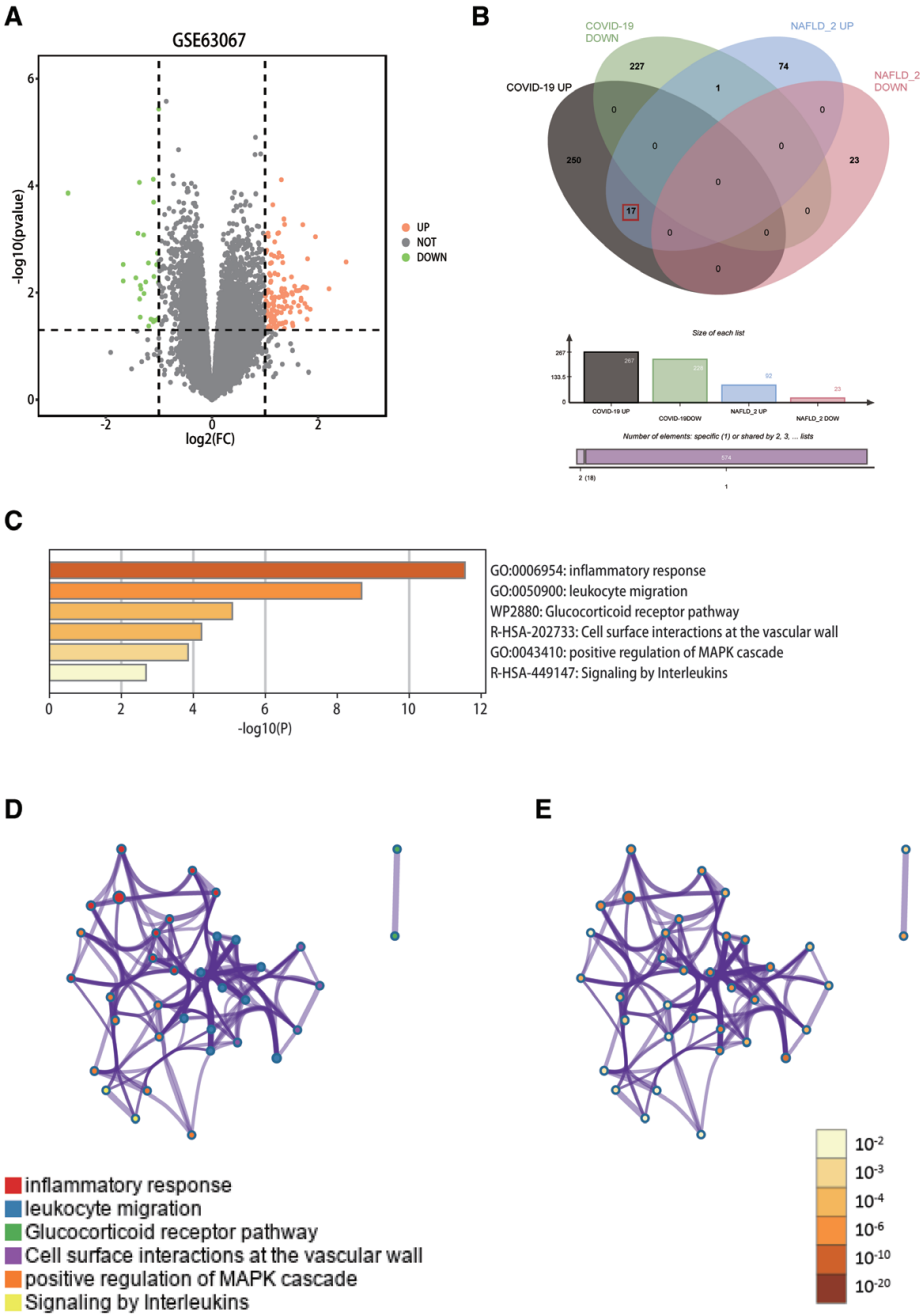
Differential gene expression and functional enrichment analysis of validation set GSE63067. (A) Differentially expressed genes (DEGs) were identified in GSE63067. (B) Co-expressed differentially expressed genes in GSE63067 and GSE147507. (C) Bar graph of enriched terms across GS2, colored by *P* values. (D, E) Network of enriched terms. (D) colored by cluster ID, where nodes that share the same cluster ID are typically close to each other. (E) colored by *P* value, where terms containing more genes tend to have a more significant *P* value.

**Figure 7.**
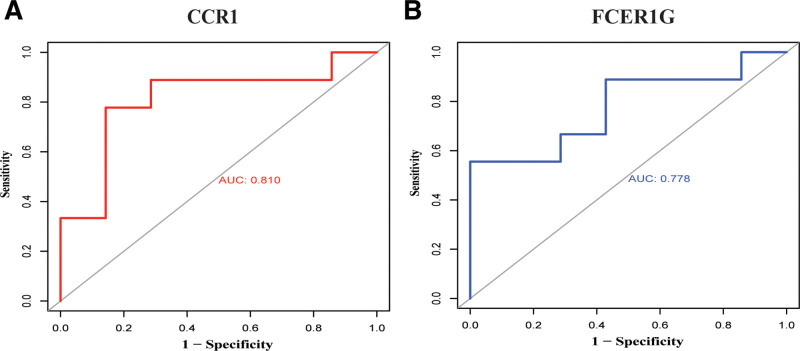
Exploring the predictive value of biomarkers. (A) ROC analysis of CCR1. (B) ROC analysis of FCER1G.

To validate our results, we performed the differential genes analysis on the GSE147507 and GSE63067 datasets, a total of 139 DEGs were identified, including 112 upregulated genes and 27 downregulated genes (Fig. 6A). Screening for valid crossover genes using Venn diagrams to identify GSE63067 and GSE147507 yielded a total of 17 common upregulated genes (Fig. 6B). GS2 was subjected to functional enrichment analysis (Fig. 6C) and GS2 is primarily involved in inflammatory, immune response, and cell signaling functions, including inflammatory responses, leukocyte migration, and positive regulation of MAPK cascade responses. To further capture the relationships between terms, a subset of enriched terms has been selected and rendered as a network diagram (Fig. 6D and E). There are 2 overlapping genes in GS1 and GS2, CCR1 and FCER1G, both of which are key genes of GS1. We performed a ROC curve analysis on CCR1 and FCER1G (Fig. 7A and B) and the results showed that AUG (CCR1) = 0.810 and AUG (FCER1G) = 0.778, resulting in a high diagnostic value. This is highly consistent with the results of the discovery cohort.

### 3.6. Identification of ferroptosis-related genes and prediction of miRNA and lncRNA

**Figure 8.**
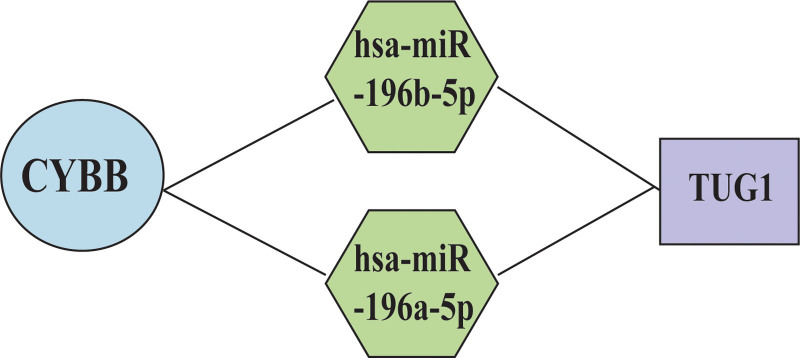
The mRNA-miRNA-lncRNA co-regulatory network associated with ferroptosis.

A total of 382 ferroptosis-related genes were obtained from the FerrDb database. Analysis of the expression of the 382 ferroptosis-related genes in the GS1 dataset identified CYBB as the differential ferroptosis gene. After that, we predicted the upstream miRNAs that might bind to CYBB, and finally identified 2 miRNAs (hsa-miR-196a-5p and hsa-miR-196b-5p), and bidirectional promoter lncRNA (TUG1). Build mRNA-miRNA-lncRNA co-regulatory network construction (Fig. 8).

### 3.7. Identification of TFs associated with Hubs

**Figure 9.**
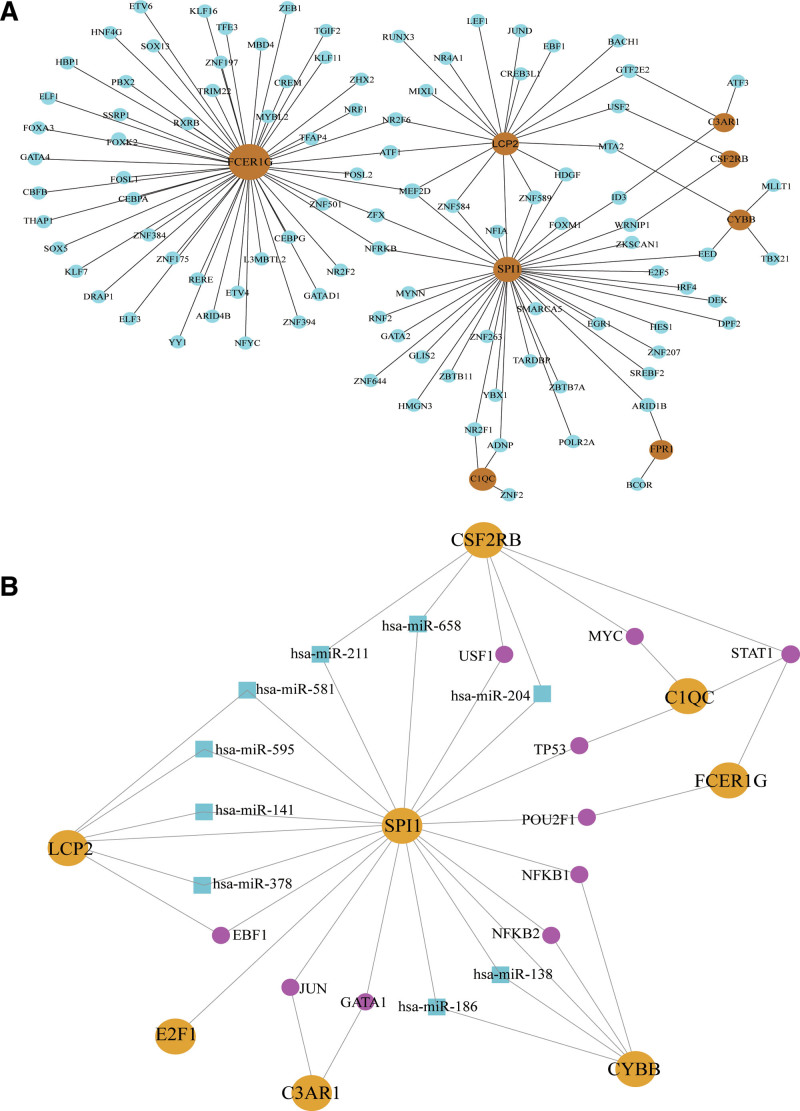
Key gene-related transcription factors (TFs) and miRNAs networks. (A) Network diagram of the interrelationship between TFs and key genes. Brown represents the key genes and blue represents TFs. (B) TFs and mi-RNAs network relationship diagram. Yellow represents the key genes, blue represents miRNAs, and purple represents TFs. TFs = transcription factors.

The top 10 key genes were imported into the Networkanalyst data analysis platform, and the results identified TFs related to 8 key genes, FCER1G, LCP2, SPI1, C3AR1, CSF2RB, CYBB, C1QC, and FPR1. Their interactions are shown in Figure 9A. The network graph consists of 108 nodes and 119 edges.

### 3.8. Analysis of TF-miRNA synergy

The Networkanalyst network platform was used to display 27 nodes and 42 edges, which consisted of 8 hub genes, 10 TF genes, and 9 miRNAs (Fig. 9B).

### 3.9. Identification of drug candidates

DSigDB is a drug signature database for gene set analysis, and the DSigDB database can be accessed through the online website (https://maayanlab.cloud/Enrichr/). We obtained 8 key genes related to TF-Gene and TF-miRNA and used DSigDB to identify the drug molecules related to them, and a total of 204 genes-drugs were identified. On the premise of meeting the set threshold *P* ≤ .05, 10 gene-targeted drugs were screened according to the comprehensive score, Eckol CTD 00002503, sulfinpyrazone TTD 00011126, phenylbutazone TTD 00010195, PCB 118 CTD 00002737, fucose TTD 00008125, sulfinpyrazone CTD 00006807, 2,2′,4,6,6′-pentachlorobiphenyl CTD 00003860, mebendazole HL60 UP, fluorometholone HL60 UP, lasalocid HL60 DOWN (Table 4).

**Table 4:** COVID-19 combined with NAFLD gene-targeted drugs.

Term	*P* value	Combined score	Genes
Eckol CTD 00002503	.004392244	1549.437088	CYBB
sulfinpyrazone TTD 00011126	.004392244	1549.437088	FPR1
phenylbutazone TTD 00010195	.005587202	1138.863728	FPR1
PCB 118 CTD 00002737	.005985243	1043.434298	CYBB
fucose TTD 00008125	.007178529	828.6537823	FCER1G
sulfinpyrazone CTD 00006807	.007973357	725.5830081	FPR1
2,2′,4,6,6′-pentachlorobiphenyl CTD 00003860	.007973357	725.5830081	CYBB
mebendazole HL60 UP	5.83E-06	692.4576092	FCER1G; C3AR1; FPR1; LCP2
fluorometholone HL60 UP	.008767628	643.5130349	FPR1
lasalocid HL60 DOWN	.008767628	643.5130349	FPR1

COVID-19 = coronavirus disease, DEGs = differentially expressed genes, NAFLD = nonalcoholic fatty liver disease.

## 4. Discussion

NAFLD is one of the most common comorbidities associated with obesity. Recent studies indicate that NAFLD is also inversely related to other chronic diseases.^[33]^ About 15% to 30% of COVID-19 patients have underlying liver disease, and 20% to 35% show liver enzyme changes on hospital admission.^[34]^ NAFLD is now considered a high risk or aggravating factor for contracting severe COVID-19 disease.^[12]^

This study used high-throughput sequencing datasets for expression profiling analysis, the sequenced gene series of the 2 diseases were integrated and analyzed with their control group gene sequences, and the relationship between NAFLD and COVID-19 was analyzed using network and computational systems. A total of 28 common DEGs were obtained and subjected to GO and KEGG pathway enrichment analyses and a PPI network was constructed to select Hub genes that could be used to identify effective therapeutics.

GO is the most comprehensive database that provides information about the function of genes and gene products, and is widely used in the life sciences.^[35]^ To better understand the function of DEGs involved in NAFLD and COVID-19, the GO database was used to perform a functional annotation analysis of DEGs that may be involved in biological processes, cellular components, and molecular functions. The analysis found that DEGs are mainly involved in biological processes such as bone marrow leukocyte activation, immune responses, neutrophil degranulation, and exocytosis. In terms of molecular functions, DEGs primarily mediate immune and complement receptor activity. Of the cellular components, DEGs are mainly enriched in the secretory granule, ficin-1-rich granule, cytoplasmic vesicle, tertiary granule, and cytoplasmic membranes. Studies indicate that immune modulation in the lung affects the severity of COVID-19 infection. In addition, both the adaptive and innate immune responses play a key role in the progression of NAFLD. Indeed, all cells involved in disease progression have an immunomodulatory function. The disruption of a stable immune regulatory mechanism alters the normal cytokine profile and results in the development of a pro-inflammatory microenvironment.^[36,37]^ Neutrophil degranulation has a major impact on lung disease progression.^[38]^ After COVID-19 infection, neutrophils can promote exocytosis of SARS-CoV-2 particles from small secretory vesicles.^[39]^ KEGG pathway enrichment is a mapping approach used to characterize the systematic functions of organisms using molecular information.^[40]^ This method showed that the DEGs identified in this study were primarily involved in osteoclast differentiation, S. aureus infection, and complement and coagulation cascade signaling pathways, among which osteoclast differentiation was the most enriched.

CYBB, FCER1G, CCR1, LCP2, CSF2RB, C3AR1, SPI1, LILRB2, C1QC, and FPR1 were the top 10 Hub genes identified by the MCC algorithm. Nonalcoholic steatohepatitis (NASH) is characterized by steatosis, liver cell damage, and inflammation and is an advanced form of NAFLD.^[41]^ CYBB can be mediated by the gene regulator TAZ in NASH hepatocytes, resulting in oxidative DNA damage, which in turn affects the progression of liver disease.^[42]^ In this study, GO and KEGG enrichment analysis showed that CYBB was related to immune response and Osteoclast differentiation. FCER1G acts as a high-affinity receptor for IgE, which induces the release of allergic response mediators and modulates allergic reactions.^[43]^ The chemokine receptor network controls immune responses and guides the migration of immune cells. CCR1 is one of the C-C chemokine receptors and has corresponding responses to a variety of inflammatory chemokines.^[44]^ Chua et al^[45]^ found that the expression of CCR1 in macrophages of critically ill COVID-19 patients was significantly increased. CCR1 can induce the recruitment of monocytes into the lung parenchyma by binding to CCL2 or CCL3 in vivo and then differentiating into inflammatory macrophages. and continuously recruit and activate other immune cells and epithelial damage, so CCR1 can be considered a potential therapeutic target. Lymphocyte cytoplasmic protein 2 (LCP2) is an actin-binding protein which activates T cells and promotes the secretion of IL-2 and IFN-γ. Changes in the quantity of LCP2 can affect immune regulatory responses.^[46,47]^ Colony-stimulating factor 2 receptor beta (CSF2RB) is a shared subunit of interleukin 3 (IL3), colony-stimulating factor 2 (CSF2) and IL5 receptors involved in signal transduction.^[48]^ CSF2RB polymorphism reduces erythropoietin-mediated NO production, abrogating the protective effect of erythropoietin on SARS-Cov-2.^[49]^ C3a receptor 1 (C3AR1) is a complement receptor that controls neutrophil extracellular trap formation and is implicated in COVID-19 disease progression.^[50,51]^ Das Deepyaman et al^[52]^ found that SPI1 or its protein product PU.1 can up-regulate the expression levels of CCL5 and IFIT3. CCL5 is activated as a pro-inflammatory chemokine during primary respiratory syncytial virus (RSV) infection.^[53]^ During influenza infection in airway and lung cells, IFIT3 can limit the spread of virus, affect the severity of infection and improve cellular defense against virus, IFIT3 may be an important mediator of influenza antiviral response.^[54]^ Therefore, it is speculated that SPI1 is involved in and amplifies the inflammatory response in the progression of COVID-19. LILRB receptor negatively regulates the activation of various immune cells (especially bone marrow cells). Leukocyte Ig-like receptor B2 (LILRB2), as a member of the Leukocyte immunoglobulin-like receptor sub-family B, is more limited to the, In addition, LILRB2 can bind to the human leukocyte antigen HLA-G and participate in the downstream expression of IL-4 and IL-13, which can not only inhibit the release of pro-inflammatory cytokines but also promote IL-10 and TGFβ to regulate cells secretion of factors.^[55,56]^ Complement C1q subcomponent C – the gene product of C1Q – and immunoglobulin kappa variable 4-1, activate the classical complement pathway and complete the immune response link.^[57]^ Formyl peptide receptor 1 (FPR1) is a G protein-coupled 7-transmembrane cell surface receptor that guides the migration of neutrophils to inflammatory lesions during sterile liver injury.^[58]^ Thus, we speculate that FPR1 plays a similar role in the progression of NAFLD.

Ferroptosis, characterized by iron accumulation and lipid peroxidation, is a newly demonstrated form of programed cell death.^[59]^ There is now evidence that ferroptosis is a major fine-tuning process of tissue damage inhibition and that they may be over-activated as dangerous stimuli in pathologies such as NAFLD/ NASH^.[60]^ At the same time, NAFLD progression may include exaggerated production of ROS and NO derivatives.^[61]^ In addition, a cohort study showed that COVID-19 disease severity was associated with serum ferritin levels as well as hyperferritinemia syndrome, while iron and ferritin were associated with ferroptosis.^[62–64]^ Therefore, we speculate that the progression of the disease in patients with 2019-nCoV infection combined with NAFLD may be related to ferroptosis. Current research suggests that when ferroptosis occurs, the Fenton reaction promotes the production of a large number of reactive oxygen species (ROS). NADPH oxidase is an important type of NOX family whose main role is the production of reactive oxygen species (ROS). CYBB is a co-regulated gene in COVID-19 and NAFLD and is also involved in encoding cytochrome b-245, which is an important component of NADPH oxidase. In addition, Zhong et al^[65]^ found through proteomic studies that CYBB can act as a ferroptosis drug target of action. Upon further study, we also predicted miRNAs and lncRNAs associated with CYBB. Therefore, we hypothesized that the ferroptosis during the progression of COVID-19 and NAFLD might be regulated through the CYBB-hsa-miR-196a/b-5p-TUG1 axis.

MiRNAs and TFs are both involved in gene regulation, interacting with each other during biological processes, and playing a co-regulatory role in gene transcription and expression.^[66]^ To explore the relationship between TF genes, miRNAs and DEGs in COVID-19 and NAFLD disease, TF-deg and TF-miRNA networks were constructed. Finally, 8 related key genes (FCER1G, LCP2, SPI1, C3AR1, CSF2RB, CYBB, C1QC, and FPR1) were obtained. In the TF-DEG network, FCER1G was highly correlated with TF gene expression. This implies that multiple TF genes can affect FCER1G expression and potentially alter disease progression. In addition, it was found from the network diagram that the transcription factor myocyte enhancer factor-2 D (MEF2D) was simultaneously associated with 3 key genes, FCER1G, SPI1, and LCP2. MEF2D plays an important role in regulating inflammatory homeostasis partly through transcriptional regulation of the type-I interferon signaling pathway.^[67]^ In the TF-miRNA network, SPI1 correlated with a high number of TF genes and miRNAs. Meanwhile, in the TF-miRNA coregulatory networks, STAT1 can regulate 3 critical genes (CSF2RB, C1QC, FCER1G).

We list a total of 10 gene-targeted drugs. Among them, Eckol and sulfinpyrazone have high binding rates to key genes. It is well known that mitochondria are rich in iron (iron is an essential ion in the mitochondrial oxidative respiratory chain) and mainly produce ROS. Therefore, it is considered to be an important site for the occurrence of iron death. Studies have shown that Eckol protects cells from mitochondrial oxidative stress by activating AMPK/FoxO3a-mediated induction of manganese superoxide dismutase (Mn-SOD), thereby reducing the expression level of ROS.^[68]^ In addition, CYBB was the target gene of Eckol by DSigDB database analysis. In the previous article, we concluded that CYBB is involved in and affects ferroptosis. Therefore, we speculate that Eckol may affect the ferroptosis process in patients with 2019-nCoV infection and NAFLD by regulating the expression of CYBB and Mn-SOD. Sulfinpyrazone is a non-steroidal anti-inflammatory drug; the anti-inflammatory activity of sulfinpyrazone is beneficial in the treatment of COVID-19 when SARS-CoV-2 causes an inflammatory cytokine storm. It was also demonstrated in a molecular docking study. Now, sulfinpyrazone has strong stability and conformational flexibility with the active site of the SARS-CoV-2 main protease.^[69]^

This study used public databases and analytic software to conduct high-throughput data analysis. Using the genetic database to find DEGs that were expressed during both NAFLD and COVID-19, the development of these diseases was shown to be primarily related to immune function and inflammation. It is speculated that NAFLD may affect COVID-19 disease progression through common signaling pathways. The mechanism of action of antimalarial drugs may be related to osteoblast differentiation. For Hub genes, TFs and miRNAs are potential targets for COVID-19 and NAFLD treatment that warrant additional study. This study did not simply investigate the mechanism of action of a single disease but explores the potential association between NAFLD and COVID-19 in a refined manner. Drug repositioning was achieved by screening potential drug molecules. These findings provide direction for future in vivo and in vitro experiments as well as clinical trials.

## Author contributions

**Conceptualization:** Wenbo Dong, Hongling Jia.

Formal analysis: Wenbo Dong.

Funding acquisition: Yan Jin.

Methodology: Wenbo Dong, Hongshuo Shi, Xuecheng Zhang.

Software: Jinshu Chen.

Supervision: Yan Jin, Hongshuo Shi, Xuecheng Zhang, Hongling Jia, Yongchen Zhang.

Validation: Wenbo Dong.

Visualization: Wenbo Dong, Jinshu Chen.

Writing – original draft: Wenbo Dong.

Writing – review & editing: Yan Jin, Hongshuo Shi, Xuecheng Zhang, Jinshu Chen, Hongling Jia, Yongchen Zhang.
